# An FE investigation simulating intra-operative corrective forces applied to correct scoliosis deformity

**DOI:** 10.1186/1748-7161-8-9

**Published:** 2013-05-16

**Authors:** J Paige Little, Maree T Izatt, Robert D Labrom, Geoffrey N Askin, Clayton J Adam

**Affiliations:** 1 Paediatric Spine Research Group, Institute of Health and Biomedical Innovation, Queensland University of Technology and Mater Health Services Brisbane, Room O718, Gardens Point Campus, 2 George Street, Brisbane, QLD, 4001, Australia

**Keywords:** Scoliosis, Finite element, Surgical forces, Correction

## Abstract

**Background:**

Adolescent idiopathic scoliosis (AIS) is a deformity of the spine, which may require surgical correction by attaching a rod to the patient’s spine using screws implanted in the vertebral bodies. Surgeons achieve an intra-operative reduction in the deformity by applying compressive forces across the intervertebral disc spaces while they secure the rod to the vertebra. We were interested to understand how the deformity correction is influenced by increasing magnitudes of surgical corrective forces and what tissue level stresses are predicted at the vertebral endplates due to the surgical correction.

**Methods:**

Patient-specific finite element models of the osseoligamentous spine and ribcage of eight AIS patients who underwent single rod anterior scoliosis surgery were created using pre-operative computed tomography (CT) scans. The surgically altered spine, including titanium rod and vertebral screws, was simulated. The models were analysed using data for intra-operatively measured compressive forces – three load profiles representing the mean and upper and lower standard deviation of this data were analysed. Data for the clinically observed deformity correction (Cobb angle) were compared with the model-predicted correction and the model results investigated to better understand the influence of increased compressive forces on the biomechanics of the instrumented joints.

**Results:**

The predicted corrected Cobb angle for seven of the eight FE models were within the 5° clinical Cobb measurement variability for at least one of the force profiles. The largest portion of overall correction was predicted at or near the apical intervertebral disc for all load profiles. Model predictions for four of the eight patients showed endplate-to-endplate contact was occurring on adjacent endplates of one or more intervertebral disc spaces in the instrumented curve following the surgical loading steps.

**Conclusion:**

This study demonstrated there is a direct relationship between intra-operative joint compressive forces and the degree of deformity correction achieved. The majority of the deformity correction will occur at or in adjacent spinal levels to the apex of the deformity. This study highlighted the importance of the intervertebral disc space anatomy in governing the coronal plane deformity correction and the limit of this correction will be when bone-to-bone contact of the opposing vertebral endplates occurs.

## Background

**Figure 1:**
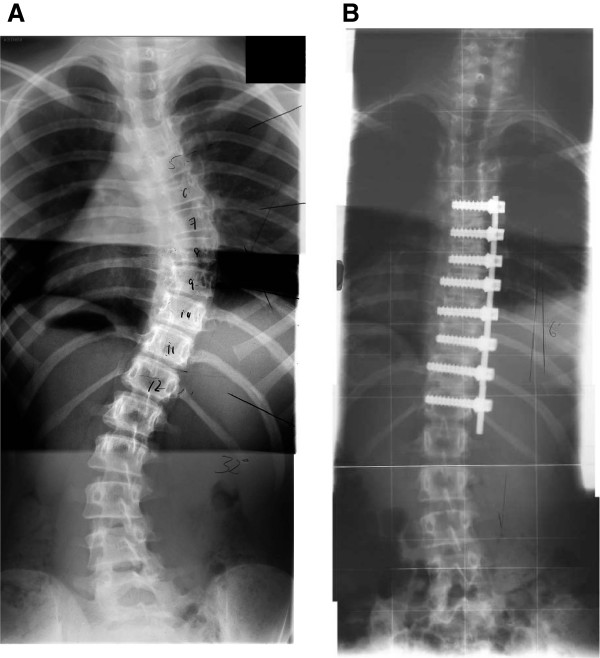
Radiographs of an AIS patient’s spine, pre-operatively (A) and post-operatively (B) after undergoing a single rod, anterior procedure.

Scoliosis is a three dimensional deformity of the spine, involving a side-to-side curvature in the coronal plane and axial rotation of vertebrae in the transverse plane (Figure 1A). Patients with severe or progressive deformities are generally treated surgically, and surgical correction aims to arrest curve progression and achieve the best possible improvement in deformity through a reduced Cobb angle, while minimizing the risk of surgical complications (Scoliosis Research Society, [1]). However despite improved 3rd generation implant designs, implant related complication rates are still high. A recent meta-analysis [2] reported an overall mean complication rate of 20% for 5,780 adolescent idiopathic scoliosis (AIS) patients who had undergone scoliosis corrective surgery.

The anterior single rod correction procedure is one possible surgical technique [3] (Figure 1B) for treating scoliosis. This procedure involves removing the deformed intervertebral discs, implanting material to promote fusion of the intervertebral joint space and securing metal rods to the spinal vertebra using screws [4]. The surgeon achieves an intra-operative reduction in the patient’s deformity by applying compressive forces across the fused intervertebral disc spaces via pairs of adjacent screws, while securing the rod to the vertebra.

Previous researchers have demonstrated the potential of computational methods [5] and in particular finite element (FE) models to investigate the mechanics of the scoliotic spine during surgery [6-8]. FE models which are personalized to include representations for the individual patient’s soft and osseous anatomy and spinal loading conditions, have the potential to assist surgeons in planning the patient’s surgery and in optimizing their treatment in order to obtain the best possible surgical outcomes. Irrespective of the aetiology of the spinal deformity, surgical treatment involves applying biomechanical corrective forces to the spine using implants attached to the spinal anatomy. Implant related complications involve mechanical failure of the spinal tissues, thus an investigation of the biomechanics of the surgically corrected spine lends itself to the use of the FE method which is able to predict stresses and strains in both implants and spinal tissues.

The aim of this study is to use FE models derived from computed tomography data for the thoracolumbar spine and ribcage of AIS patients, to investigate the biomechanics of the surgically corrected spine during single rod anterior scoliosis surgery. The question of interest is how the AIS deformity – specifically the coronal Cobb angle and disc space deformity - is reduced with increasing magnitudes of surgical corrective force. The ability of the FE model to predict tissue-level stresses was also used to predict the surgically induced contact stresses between adjacent vertebral endplates.

## Methods

FE models for eight AIS patients who underwent single rod thoracoscopic anterior scoliosis surgery (Table 1) were individualized to the patient’s osseous anatomy using clinically indicated, low-dose (2.0-3.7 mSv radiation dose) computed tomography (CT) scans. These CT scans were obtained pre-operatively for surgical planning purposes [9]. This study involved a retrospective investigation of FE simulated outcomes for this series of patients, who had previously been treated at the Mater Children’s Hospital in Brisbane, Australia. In order to determine the effect of varying surgical corrective forces on the predicted deformity correction, these models were analysed using statistical data for intra-operative compressive forces measured in a recent experimental study by our group [10]. The FE models were analysed using ABAQUS 6.9-1 (Dassault Systemes, France) on an SGI Altix XE computational cluster (608 × 64 bit Cores, 1728 GB memory).

**Table 1:** Patient demographics for AIS patients

**Patient**	**Age**	**Height (cm)**	**Weight (kg)**	**Gender**	**Pre-operative major Cobb angle (degrees)**	**Post-operative major Cobb angle (degrees)**	**Instrumented vertebral levels**	**Disc at the apex of the curve**
1	14	157	39.5	F	52	23	T5-T11	T8T9
2	21	163	49	F	51	18	T7-L1	T10T11
3	14	165	65	F	44	14	T5-T12	T8T9
4	14	157	77.4	M	53	25	T5-T12	T8T9
5	14	161	45.7	F	40	10	T5-T10	T6T7
6	23	171	61.7	F	42	7	T5-T12	T8T9
7	18	172	61.7	F	42	13	T5-T11	T7T8
8	14	161	84.7	F	53	34	T5-T11	T7T8

### Patient-specific anatomy and finite element (FE) models for AIS patients

Our method for generating three-dimensional patient-specific osseo-ligamentous anatomy and FE model geometry for the thoracolumbar spine and ribcage has been described elsewhere [8,11], so will only be briefly presented here.

**Figure 2:**
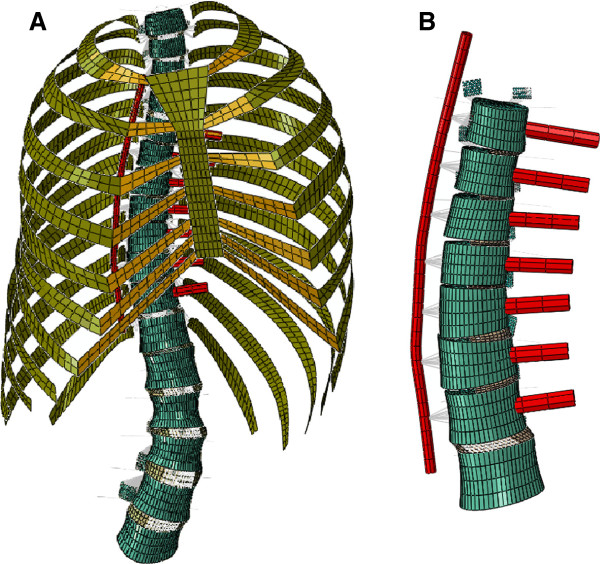
**Full spine FE model (A).** The intact thoracolumbar spine FE model for patient one; (**B**). The surgically altered thoracic spine, showing screws inserted in the vertebral bodies at the levels which were instrumented clinically for this patient and the remaining intervertebral disc portion at the intermediate disc spaces. (Note the screws are shown with extended length for visualization).

Using custom-developed algorithms and image-processing software (Matlab R2007b, The Mathworks, Natick, MA) the co-ordinates for specific bony landmarks on the vertebrae, ribs and sternum/manubrium were manually selected from the thresholded CT datasets. These landmarks were imported into custom FE pre-processing software written in Python (Python 2.5, Python Software Foundation) which used parametric descriptions for the vertebral bodies and posterior bony elements, ribs (including costal cartilage), sternum, intervertebral discs (nucleus pulposus and collagen fibre-reinforced anulus fibrosus), facet joints and ligaments to create an osseo-ligamentous FE model of the thoracolumbar spine and ribcage (Figure 2A) with anatomy personalized to the individual patient. Seven spinal ligaments were simulated at each vertebral level and were represented as either linear connectors or in the case of the anterior/posterior longitudinal ligaments, spring elements in series and parallel. The ligaments were defined between bony attachments, with no ligament wrapping simulated.

Three-dimensional geometry for the vertebral bodies was interpolated between the vertebral endplates [12] and similarly, the intervertebral disc geometry was interpolated from the adjacent vertebral endplates*.* The curved transverse profile for the articulating surfaces of the facet joints was described using a sinusoidal curve and the curvatures of the ribs were defined using 5th order polynomials, with both derived from user-selected bony landmarks. Interfacial contact between the articulating surfaces of the facet joints was modelled using exponential softened contact (normal contact) and zero-friction tangential sliding.

The costo-vertebral joints were represented in detail, since these structures are of key importance in governing the biomechanics of the spine [13,14]. Both the costo-vertebral and costo-transverse connections were represented and our method for simulating this joint has been described and validated in a previous study [15].

The elements and material parameters describing the simulated spinal structures are detailed in Table 2. There is a paucity of experimental data describing the mechanical behaviour of adolescent spinal tissues, much less tissues from AIS patients. The two main methods available to determine these properties are either direct measurement, which is highly challenging due to difficulties in accessing the tissues and obtaining precise biomechanical data, or inverse determination using data from pre-operative flexibility assessments carried out by the patients. A recent biomechanical study measured the three dimensional load–displacement response across spinal joints for two AIS patients [16] using a highly innovative technique to obtain *in vivo* joint stiffness. However, the results of this study are preliminary with data for only two patients and using this technique, the resulting joint stiffness does not provide sufficient resolution to determine individual tissue behaviour. In our previous studies we have attempted to derive patient-specific soft tissue parameters for the spinal ligaments using clinical data from pre-operative flexibility assessments [17,18]. In these studies, due to the lack of literature for the adolescent tissues, an initial set of ‘benchmark’ tissue parameters were based on the mechanical properties derived from adult subjects (Table 2) [19-30].

**Table 2:** Details of the element types and material parameters (with references) included in the FE models

**Anatomical structure**	**Element type**	**Material parameters**	**References**
Vertebral body			
- Cortical shell	4-node shell	Linear elastic E = 11,300 MPa, ν = 0.2	[25]
- Cancellous bone	8-node brick	Linear elastic E = 140 MPa, ν = 0.2	[25]
Vertebral posterior elements	2-node beam	Quasi-rigid	
Facet joints	4-node shell	As for cortical bone, with exponential softened contact between adjacent facet surfaces	
Intervertebral discs			
- Anulus fibrosus	8-node brick	Hyperelastic, Mooney-Rivlin C_10_ = 0.7, C_0_1 = 0.2	[24,26]
- Collagen fibres	Tension-only, ABAQUS ‘rebar’ elements	Linear elastic E = 500 MPa, ν = 0.3	[22]
- Nucleus pulposus	4-node, hydrostatic fluid	Incompressible	[26]
Ribs	4-node shell	Linear elastic E = 9,860 MPa, ν = 0.3	[21]
Costal cartilage	4-node shell	Linear elastic E = 49 MPa, ν = 0.4	[21]
Sternum/Manubrium	4-node shell	Linear elastic E = 9,860 MPa, ν = 0.3	[21]
Costo-vertebral joints	2-node beam	Linear elastic E_compr_ = 245 N/mm; Torsional stiffness, k_t_ = 4167Nmm/rad; Bending stiffness, k_b_ = 6706Nmm/rad (average antero-posterior and cranio-caudal flexion stiffness)	[19,23]
Ligaments			
- Ligamentum flava, supra-/inter-spinous, capsular, inter-transverse	2-node, tension-only connector	Piecewise, non-linear elastic	[20,27,30]
- Anterior/posterior longitudinal ligament	2-node spring	Piecewise, non-linear elastic	[27]
- Inter-costal connections	2-node, tension-only connector	Linear elastic, E = 25 MPa	[28]
Implant			
- Screws	8-node brick	Linear elastic, E = 108,000 MPa, ν = 0.3	Titanium alloy
- Rod	8-node brick and 2-node rigid beam	Linear elastic, perfectly plastic E = 108,000 MPa, ν = 0.3 Yield Stress = 390 MPa	Titanium alloy

### Modelling the surgically altered spine

The eight patients modeled in the study had previously undergone a single rod, anterior scoliosis procedure and clinical follow-up data was available. In carrying out this procedure the surgeon produces an immediate (intra-operative) reduction in the spinal deformity by attaching a metal rod to the anterior spinal column. The surgery firstly involves the insertion of screws into the vertebral bodies within the primary structural curve. Screws are inserted on the convex side of the primary structural curve, directly into the lateral side each vertebral body. Following this, the discs within the limits of this curve are partially removed (within the limits of endoscopic surgical accesss) and the disc spaces are packed with bone graft to promote bony fusion after surgery. In a step-wise manner, the surgeon then applies compressive forces between the screw heads of adjacent pairs of vertebrae (starting at the most-caudal motion segment in the structural curve), to reduce the level-wise deformity at each motion segment, and then locks the screws onto the rod. Thus, these level-wise corrections produce a cumulative reduction in the overall spinal deformity, which is held in place by the screw heads being locked onto the rod.

This surgical procedure was simulated for the eight patients included in this study, by adding the screws and rod to each patient-specific model, and by removing disc material from the models in the same manner as the surgically performed discectomies. Clinical data for the surgical procedure carried out on each patient was used to simulate the surgery in each patient FE model – the portion of intervertebral disc elements removed from each simulated joint space was representative of the amount of disc material extracted clinically; the clinical spinal levels fused were used to define the vertebrae in which screws were simulated; and the geometry for the simulated surgical instruments was representative of the screw diameters and rod diameter implanted for each patient. As such, eight patient-specific surgically altered spines were simulated. The screws were assumed to be perfectly bonded to the surrounding vertebral bone, so the contact mechanics of the bone-implant interface was not considered in the models. In modelling the discectomies, the fused intervertebral levels were simulated by removing approximately two-thirds of the brick elements representing the anulus fibrosus and by removing the entire hydrostatic fluid cavity representing the nucleus pulposus for these discs. The bone graft material was not simulated in this study, since bony fusion does not occur until 3–6 months after surgery and the material offers minimal mechanical resistance during surgery. The contact interaction between the exposed vertebral endplates at the discectomy levels was modelled using an exponential, softened contact relationship for normal contact and Coulomb friction (μ = 0.3) for tangential sliding. This softened contact relationship simulated a cartilaginous endplate thickness of 0.1 mm, being the distance at which the contact pressure between adjacent endplates became non-zero.

### Simulating the intra-operative loadcase and boundary conditions

#### Surgical forces

There is limited biomechanical data available in the literature describing the surgical forces applied intra-operatively during anterior spinal deformity surgery. As such, in a recent *in vivo* biomechanical study by members of our group, Fairhurst et al. [10] presented intra-operatively measured force data for a series of 15 AIS patients who underwent the single rod anterior corrective procedure. This study presented descriptive mechanical data (mean and standard deviation) for the surgical corrective forces applied intra-operatively at each intervertebral level, normalized by vertebral level relative to the apex of the curve. (This study was performed with approval from the Mater Children’s Hospital Ethics Committee). Due to the timing of the two studies, the 15 patients in this previous biomechanical study were not included in the patient series for the current study. While these biomechanical data could not be used to provide personalized force data for the eight AIS patient FE models in the current study, the data was still invaluable in providing representative values for intra-operatively applied forces in a comparable patient data set – data which was heretofore unavailable in the literature.

In keeping with the study by Fairhurst et al. [10], if the apex of the deformity for the patients in the current study was a vertebra (eg. T7), the disc space caudal to this was defined as the apical level (T7T8). Using these data for the mean and standard deviation, three separate compressive force profiles were defined in the current study (Table 3) and these forces were applied to the patient-specific models by normalizing the structural curve in each model using the same method presented by Fairhurst et al. [10]. The three different force profiles were used to investigate the sensitivity of the predicted deformity correction to intra-operative surgical forces.

**Table 3:** **Three separate force profiles simulated for each patient-specific FE model - based on the mean and standard deviation *in vivo *measurements of Fairhurst et al. (2011)**[10]

**Normalized vertebral level**	**Force profile A (Mean + SD, [**[10]**])**	**Force profile B (Mean, [**[10]**])**	**Force profile C (Mean – SD, [**[10]**])**
−3 (Superior-most vertebra)	580	400	230
−2	765	580	395
−1	895	675	455
0 (Apical Disc)	945	660	380
+1	750	550	355
+2	635	470	300
+3 (Inferior-most vertebra)	495	320	145

Note that all forces are in Newtons.

#### Boundary conditions

To simulate the guided sliding movement of the screws along the rod during surgery, a ‘no separation’ normal contact and frictionless tangential contact definition were defined between the screw head and the surface of the rod. After the surgical force loading step had been applied for each pair of adjacent screws, this tangential contact definition was changed to roughened (bonded) contact to simulate the surgical procedure for locking the screws onto the rod. During the simulations the spine was fully constrained from rigid body motion at the inferior-most vertebral level (L5) and stabilized in the lateral direction at the superior-most vertebra to simulate the constraint provided by the operating table (since the patient is positioned in the lateral decubitus position on the operating table).

#### Rod pre-bend

Intra-operatively, the rod is pre-bent manually prior to being attached to the vertebrae [4]. The angle of rod pre-bend varies from patient to patient based on the surgeon’s judgement of the achievable deformity correction and is not measured clinically. In the absence of measured values for the rod pre-bend angle in each case, the simulated pre-bend in the models was based on the pre-operative coronal Cobb angle for each patient. In this study, a ‘prebend’ load step was performed in which the screw heads were fixed in space, and then the connector elements between the rod and the screw heads were reduced to zero length (these elements provide an axial link between the connected nodes on the rod and screw and have no associated stiffness), in order to bend the rod to conform to the pre-operative profile of the spine. Following the prebend load step, the fixed boundary constraint on the screw heads was removed in the second loadstep allowing elastic springback of the rod prior to the actual surgery simulation steps.

### Analysis

As described above, each of the eight patient-specific models were analyzed using three separate intra-operative force profiles (Force profiles A, B and C in Table 3). These 24 analyses were performed using a quasi-static solver (no inertial effects) with the ABAQUS nonlinear geometry capability enabled.

The predicted corrected Cobb angle for the instrumented curve was calculated for each analysis and compared with the clinically measured post-operative Cobb angle for each patient (using the 1 week post-operative standing x-ray). In comparing model predictions with clinical measurements, the accepted clinical radiograph measurement variability of ±5^o^, [31] was taken into account.

**Figure 3:**
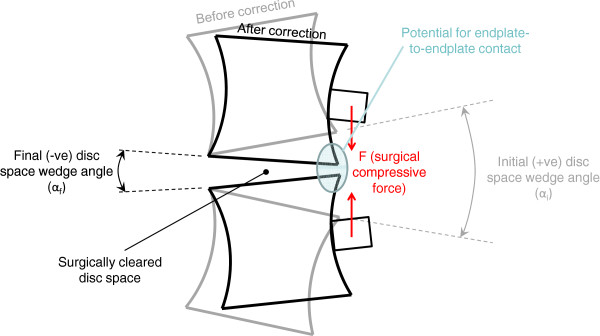
**Schematic showing an intervertebral disc space in the coronal plane, depicting the change in disc space wedge angle due to a surgical compressive force, F.** In this schematic, the surgically cleared disc space is initially wedged in the same sense as the overall spinal Cobb angle (positive wedge angle, α). As a result of the surgically applied compressive force (and depending on the stiffness of the spinal tissues), the disc space may remain positively wedged (reduced value of α, not shown), may become negative (concave wedge angle) or close the disc space entirely, resulting in endplate to endplate contact.

Since the intervertebral discs are the primary spinal structures which impart flexibility to the anterior column, anterior surgical correction of the spinal deformity primarily involves reduction of these disc spaces (height and/or wedge angle). To better understand how correction of the scoliosis deformity is achieved, the predicted change in disc space wedge angle in the coronal plane was calculated during each of the simulated surgical procedures (Figure 3). For each model, the segmental correction (ie. change in disc space wedge angle, ∆angle = α_initial_ - α_final_, Figure 3) at each intervertebral disc space was expressed as a fraction of the total correction of the instrumented curve (referred to as Disc Space Correction Ratio), in order to determine the relative contribution of each disc space to the total coronal plane Cobb angle correction. Note that a positive wedge angle α represents an angle which is opening towards the convex side of the curve, while a negative α represents a wedge angle which opens towards the concavity of the curve. The simulated contact pair separation (normal distance between adjacent endplate surfaces), and also, in cases where the endplates were in contact, the pressure between adjacent pairs of endplates, were analysed at each of the intervertebral disc spaces in the instrumented spine. From this data it was determined whether the disc space wedge angle was closing/becoming negative (ie. non-zero contact pressure, Figure 3) or whether adjacent endplates were touching (i.e. contact separation indicated bone-to-bone contact, Figure 3) due to the simulated surgery. A non-zero contact pressure in the absence of bone-to-bone contact occurred when the endplates were closing, but the normal separation distance between adjacent endplates was within the separation range (0–0.1 mm) defined using the softened-exponential contact relationship.

## Results

### Overall and segmental coronal Cobb correction

**Figure 4:**
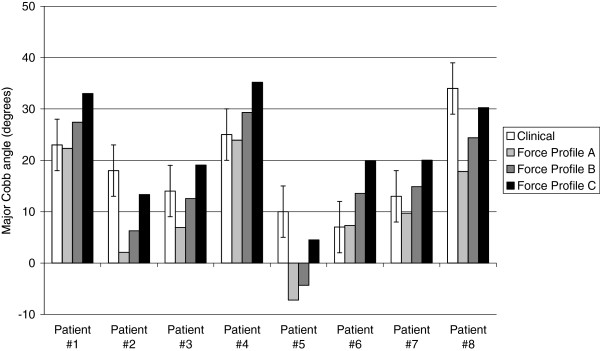
**Clinical and predicted (Force profiles A, B and C) corrected Cobb angle (degrees) for the eight patient FE models.** Error bars for the clinical Cobb angle represent ± 5^o^ variation in clinical measurements [31]

**Figure 5:**
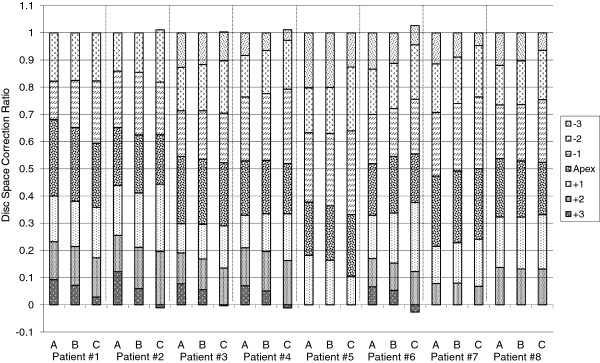
**Normalized disc space correction for each spinal level within the instrumented curve.** (The disc space level was normalized relative to the apical disc, so that the eight models could be compared.) A denotes force profile A, B denotes force profile B and C denotes force profile C. (Note that a negative correction ratio indicates the joint space wedge angle had increased compared to the pre-operative angle, however, for patients 2,3,4 and 6, this increase was less than 0.5 degrees, indicating the disc space angle was essentially the same after the simulated surgery).

The predicted results for post-operative Cobb angle for seven of the eight patient-specific models were within the 5° clinical Cobb measurement variability (Figure 4) for at least one of three force profiles. The predicted corrected Cobb angle for patient five was negative, indicating the applied corrective forces ‘over-corrected’ the spinal deformity for force profiles A and B. For all patients, there was an increase in the predicted corrected Cobb angle with increasing intra-operative compressive force. When comparing the predicted normalized disc space corrections for each of the three load profiles, the largest proportion of overall correction occurred at the apical intervertebral discs for force profiles A and B, with diminishing correction caudal and cephalic to this level (Figure 5). For Force profile C, the largest proportion of correction was observed at the apical disc in three of the eight patients, and in either the cranial or caudal peri-apical disc for the remaining five patients (Figure 5).

**Figure 6:**
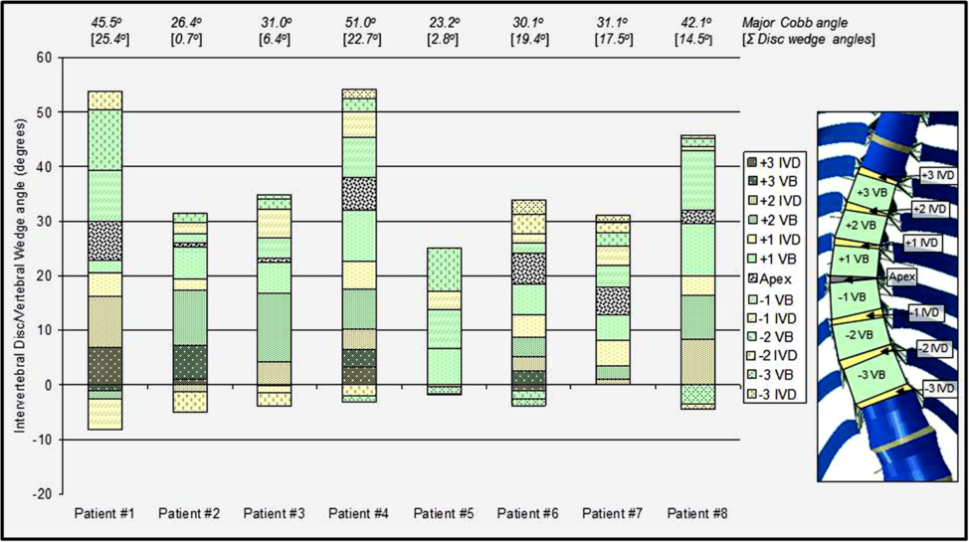
**The pre-operative coronal wedge angle for both the vertebra (green) and intervertebral discs (yellow), shown cumulatively for each patient.** Note the sum of the vertebra and intervertebral disc wedge angles for all spinal levels in a particular patient gives the overall pre-operative coronal Cobb angle (Major Cobb angle shown above the bar). Note also that negative wedge angles mean the disc or vertebra was wedged in the opposite direction to that of the major curve.

Although the largest portion of overall correction was predicted at or near the apical intervertebral disc as presented in Figure 5, this was not necessarily the disc with the largest pre-operative wedge angle (Figure 6). A comparison of the vertebral and intervertebral disc wedge angles in the coronal plane based on the model geometry before and after the surgical correction showed that between 2.6% and 64.5% of the initial coronal deformity (Cobb angle) was due to wedging in the intervertebral discs (Figure 6).

**Figure 7:**
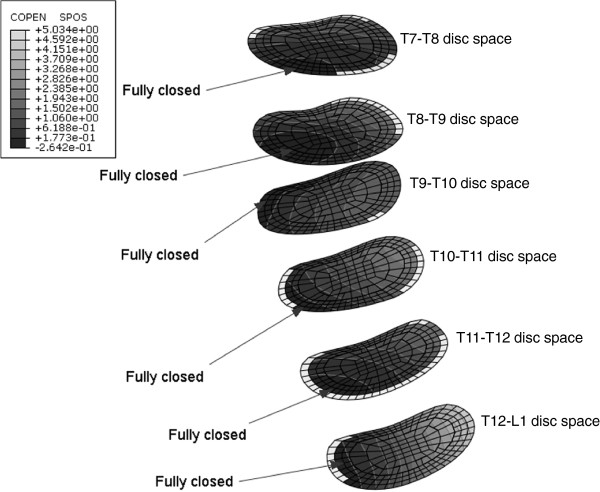
**Contact separation (distance between contacting surfaces, mm) on the inferior endplate at each intervertebral disc space (NB. For clarity of visualizing the contact distribution, the superior endplate is not shown).** A positive contour value indicates the endplates are open (white – grey); a negative contour value indicates the endplates are closed (black). The white bands on the edge of an endplate indicate disc spaces where the superior vertebra has displaced laterally compared to the inferior vertebra (ie. overhangs), thus the endplates are no longer in contact.

Model predictions for patients one, two, three and five showed endplate-to-endplate contact was occurring on adjacent endplates of one or more intervertebral disc spaces in the instrumented curve at the end of the surgical loading steps. For these disc spaces, the disc wedge angle was negative (Figure 3). Note, the initial coronal deformity for patients two, three and five was a result of primarily vertebral wedging rather than disc wedging (Figure 6). For all except patient one, with increasing corrective forces the number of intervertebral disc spaces with contact pressure on the endplates increased (eg. Patient 2, total of six disc spaces – contact pressure on six disc spaces due to Force profile A, contact pressure on three disc spaces due to Force profile B, contact pressure on one disc space due to Force profile C). The analysis for patient two predicted contact between adjacent endplates for all cleared disc spaces in the instrumented curve due to force profile A (Figure 7).

**Figure 8:**
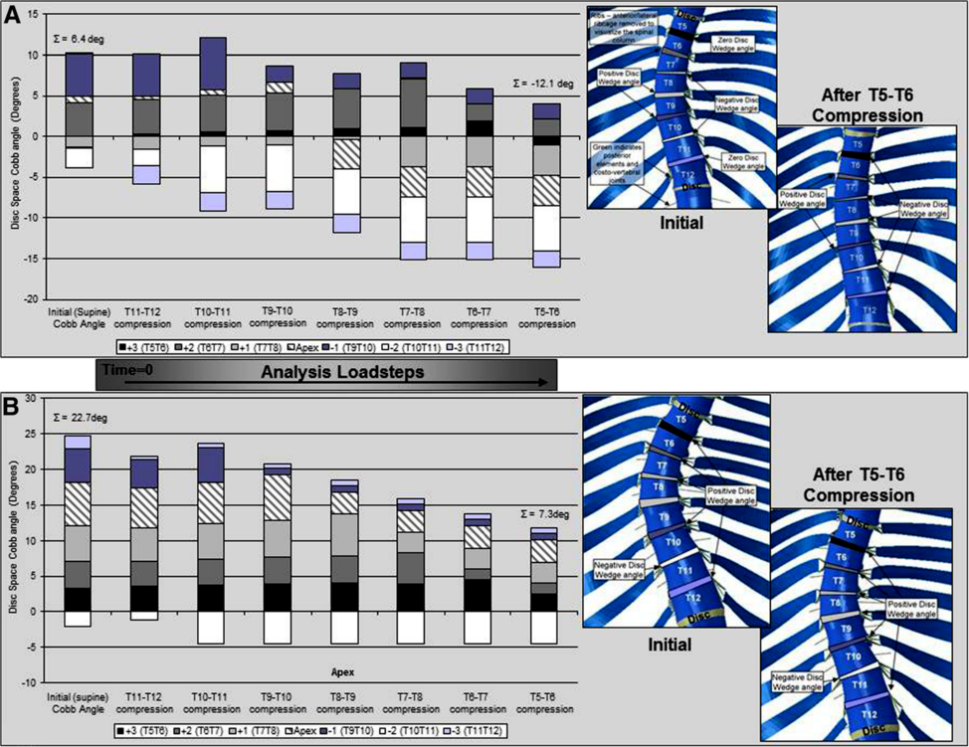
**Change in intervertebral disc space wedge angle during the simulated surgical steps for Force profile B; (A). Patient three, (B). Patient four.** Note the Σ values represent the cumulative sum of the disc wedge angles at the beginning and end of the analysis and equate to the portion of the overall coronal Cobb angle due to disc wedging. The schematics show an anterior view of the spinal column for each patient, with the disc wedge angles delineated according to the legend for the bar-chart, highlighting positive, negative and zero disc wedge angles. (Note that the ordering of the disc wedge angles in the stacked bars does not reflect the anatomical ordering in the spinal column since in some cases adjacent discs have oppositely signed wedge angles.)

Collating the results for the cumulative change in intervertebral disc space wedge angle during the level-by-level compression steps of the simulated surgery demonstrated two typical response patterns (an example of each is shown in Figure 8). Firstly, in the case of patients two, three and five, the initial cumulative disc wedge angles for these patients accounted for only 2%, 20% and 12%, respectively of the initial major Cobb angles (Figure 6, angles given above bars in chart). During the simulated surgical steps, the wedge angle in the disc spaces was progressively reduced, resulting in a cumulative reduction in the overall Cobb angle in which thefinal coronal wedge angle for the majority of the disc spaces was negative (α as described in the Methods section) (Figure 8A). Secondly, in the case of the remaining patients (one, four, six, seven and eight), the majority of the disc spaces initially demonstrated a positive wedge angle (α as described in the Methods section) which was progressively reduced with each simulated surgical load step to result in a cumulative reduction in the overall Cobb angle, however the final coronal wedge angle for the majority of the discs was still positive (Figure 8B).

## Discussion

Using patient-specific FE models of the osseoligamentous thoracolumbar spine, this study investigates the biomechanical response of eight AIS patients to surgical corrective forces applied during single rod, anterior scoliosis surgery. Each FE model was subjected to three corrective force profiles in the range of experimentally measured values, and the resulting model response was investigated with particular focus on the predicted coronal plane correction occurring in the intervertebral disc spaces following partial discectomy and single anterior rod instrumentation.

A limitation of this study is that the passive osseo-ligamentous models of the spine and ribcage used herein do not provide biomechanical insights on the response of the spine to post-operative loading conditions which involve muscle activation. While the spinal muscles may play a role in passively resisting loads applied to the spine while the patient is anaesthetized [32], the current study assumes this contribution to spinal flexibility is minimal in comparison to that of the ligamentous and cartilaginous tissues of the spine.

Post-operatively, the corrected Cobb angle is normally measured clinically using standing radiographs obtained one week after surgery. However, the comparison between the clinical and predicted corrected Cobb angle in the current study (Figure 4) was based on model predictions which were analysed for the surgical loadcase only, thus assumed the patient was still supine. Ideally, supine radiographs obtained immediately after surgery, while the patient is still recovering and so is not yet load-bearing, would provide a better clinical comparison for the predicted post-operative Cobb angle from the patient-specific models. However, these radiographs were not available for the patients in the current study. Once the rod is surgically attached to the vertebra, it is reasonable to assume that the instrumented region of the spine would experience only small intervertebral motions (< 1^o^), since the main purpose of the surgery is to ensure that motion is sufficiently restricted such that bony fusion can occur between adjacent vertebral bodies. Therefore, the difference in the clinically measured corrected Cobb angle from supine compared to standing radiographs is not expected to be of the magnitude which is observed prior to surgery in the uninstrumented spine.

The use of tissue mechanical properties derived from adult data to simulate adolescent spinal tissues is another limitation of the study, and is a necessary consequence of the paucity of paediatric and adolescent tissue mechanical data available in the literature. However, we note that tissue stiffness (e.g. the force-displacement for a whole ligament) is a result of both the inherent mechanical response of the ligament tissue itself, and the dimensions (in this case length and cross-sectional area) of the ligament. By including patient-specific anatomical landmarks as the ligament attachment points in the models, the patient-specific modeling approach used in the current study incorporates variations in ligament length between patients, and therefore goes some way to simulating patient-specific tissue properties.

Another limitation of the current study is that the angle of rod prebend which is introduced prior to attaching the rod to the patient’s spine is not measured clinically and is based on the surgeon’s judgement. In the current study, this angle of prebend for the simulated surgery was estimated using the pre-operative Cobb angle, however, future studies using this patient series will focus on investigating the sensitivity of model predictions to the prebend angle and plastic prestrain in the rod.

With regard to model validation, Figure 4 showed that the predicted Cobb angles after surgical correction were within 5° agreement with the clinical values for seven of the eight patients in the study. However it is important to keep in mind that the surgery force profiles used in the study were not ‘patient specific’, since average surgery force data for an experimental measurement series [10] were used to define the model load profiles for all eight patients in the current study. The results from the current study show that increasing the simulated intra-operative forces, resulted in a reduction in the predicted corrected Cobb angle.

Measurement variability from clinical radiographs results in a wide range of error (±5^o^), and furthermore, there was large variability in the intra-operatively measured surgical forces which resulted in a similarly wide range of variation in the predicted corrected Cobb angle. It should be noted that the inter-relationship between these sources of variability may have the potential to obscure patterns in the predicted outputs. For instance, the results for patient five suggest that the average surgery forces applied to the model were higher than those applied intra-operatively for this patient. While the descriptive data for surgical forces were measured for a series of AIS patients from the same study population as patients simulated in the current study, the use of intra-operative force data measured for each individual patient would provide a more ideal simulation for individual patient loading. This reflects a limitation of the future clinical application of patient-specific modeling approaches for all such virtual spine models, in that patient-specific surgically applied forces can only be measured at the time of surgery, therefore actual patient force data can only be simulated retrospectively post-surgery. Aside from modeling considerations, the substantial variation in surgically applied corrective forces warrants further study, and there may be a case for developing technology to provide force feedback to surgeons during implant insertion.

The results of this study highlight the importance of the intervertebral disc space anatomy in governing the coronal plane deformity correction which may be achieved in the instrumented curve. Since the partially cleared intervertebral disc spaces are the primary anatomy in the anterior column of the spine imparting flexibility, the maximum correction which may be achieved surgically will be governed by the anatomy of the discs in terms of disc wedge angle and disc height. The limit of this achievable deformity correction will be when bone-to-bone contact of the opposing vertebral endplates occurs, and for different patients, this limit will be achieved with varying magnitudes of surgical corrective forces. One of the strengths of the patient-specific model geometry used here is the ability to capture endplate to endplate contact during the surgical correction, and thus to predict the diminishing return between applied corrective force and segmental correction.

Results for the predicted corrected Cobb angle indicate that there is an inverse relationship between the magnitude of the total corrective force and the decrease in corrected Cobb angle and this is a proportional relationship for all except patient two. By increasing the total corrective force by as much as 120% (comparing the total force applied in Profile A to the total for Profile C), this resulted in a reduction in the corrected Cobb angle. For example, for patient three, the corrected Cobb angle for Profile C was 19.1^o^ and for Profile A was 6.9^o^ (Figure 4), which represented a 64% reduction in the corrected Cobb angle with increasing corrective force. This percentage reduction in corrected Cobb angle ranged from 32 to 84% when comparing the results for Profile A to Profile C for the eight patients (Figure 4). Moreover, as stated above, the anatomy of the discs will strongly influence the maximum achievable correction and for some patients, applying increasing magnitude corrective forces will result in bone-to-bone contact in the disc space and unnecessarily load the vertebral bone with a comparatively minor improvement in deformity correction. As such, the interaction of these key biomechanical factors of force, geometry (patient anatomy) and tissue stresses is of key importance in achieving an optimal correction for a patient, with the least risk of excessive loads on the spinal tissues causing possible implant related complications. Herein lies a key advantage of use of patient-specific FE models as tools to assist surgeons in pre-operative planning for deformity surgery.

## Conclusions

Attempts to improve the outcomes of spinal deformity surgery using patient-specific computer models depend strongly on the ability of the models to correctly capture the anatomy, tissue mechanical properties, and applied loading in individual patients for their validity. The simulations presented in this study are an initial step in the development of computational tools to predict surgical deformity correction. This study demonstrated a direct relationship between the surgically applied corrective forces and the deformity correction achieved, showing that the majority of deformity correction occurs in the intervertebral disc spaces at or near the apex of the deformity. The study results highlighted the importance of the intervertebral disc space anatomy in influencing the coronal plane deformity correction. By better understanding how the mechanics of a patient’s spine is altered during scoliosis corrective surgery, patient-specific models such as these can potentially provide an improved understanding of how to achieve an optimum correction for an individual patient’s spine.

## Competing interests

The authors declare thay they have no competing interests.

## Authors’ contribution

JPL and CJA were responsible for the conception of the study and carried out the computational analysis and data postprocessing for the study. MI collated and analysed the clinical patient data for study. GNA and RDL performed the surgical procedures and provided instructions and advice on how to replicate the surgical procedure computationally. All authors read and approved the final manuscript.
